# A Novel Concrete-Based Sensor for Detection of Ice and Water on Roads and Bridges

**DOI:** 10.3390/s17122912

**Published:** 2017-12-14

**Authors:** Habib Tabatabai, Mohammed Aljuboori

**Affiliations:** Department of Civil and Environmental Engineering, University of Wisconsin, Milwaukee, 3200 N Cramer St, Milwaukee, WI 53211, USA; aljuboo2@uwm.edu

**Keywords:** concrete, black ice, safety, ice detection, bridge decks, pavements, electrical resistance

## Abstract

Hundreds of people are killed or injured annually in the United States in accidents related to ice formation on roadways and bridge decks. In this paper, a novel embedded sensor system is proposed for the detection of black ice as well as wet, dry, and frozen pavement conditions on roads, runways, and bridges. The proposed sensor works by detecting changes in electrical resistance between two sets of stainless steel poles embedded in the concrete sensor to assess surface and near-surface conditions. A preliminary decision algorithm is developed that utilizes sensor outputs indicating resistance changes and surface temperature. The sensor consists of a 102-mm-diameter, 38-mm-high, concrete cylinder. Laboratory results indicate that the proposed sensor can effectively detect surface ice and wet conditions even in the presence of deicing chlorides and rubber residue. This sensor can further distinguish black ice from ice that may exist within concrete pores.

## 1. Introduction 

Surface ice (also referred to as black ice or glaze ice) is a thin layer of frozen water that can form on the roadway surface. This transparent layer is commonly referred to as black ice because the pavement surface can be seen while the ice may not be visible. Black ice can typically form when moisture (from rain, fog, etc.) comes in contact with a surface that has a temperature below freezing. Black ice affects aircraft on runways and taxiways, cars and trucks on the roads, and pedestrians and cyclists on sidewalks or walkways. As a layer of ice forms on a surface, the contact friction decreases drastically. This condition significantly increases the slippage hazard for pedestrians and vehicular traffic. 

Surface ice needs two main components to form: moisture and low temperatures. There are many potential sources of moisture for the formation of black ice such as rain, snow, hail, accidental water discharge, sleet, freezing fog, or blowing and drifting snow [1]. Black ice can sometimes form when there is a sudden warm-up after a long period of very cold weather. The pavement remains below freezing, while there is moisture available in the air above the surface [1]. Formation of surface ice occurs more frequently on bridge decks because the decks are exposed to air circulation on both their top and bottom surfaces.

According to www.icyroadsafety.com, black ice causes more weather-relevant deaths and injuries annually than all other severe weather conditions combined [2]. Ice hazards differ from location to location, and may depend on roadway alignment. For example, if black ice forms on a downhill surface, the potential for slip increases. The micro- and macrotexture properties of the pavement surface have an important influence on long-term safety, especially in cases when surface treatments (chip seals, thin polymer overlays, etc.) are used [3,4,5].

Current methods for the detection of ice on roadway surfaces include several non-embedded and embedded sensing systems. Non-contact sensing systems typically employ infrared technologies and use cameras that are mounted on elevated supports to assess their surrounding areas [6,7]. There are also vehicle-mounted systems including a system that estimates the difference in speed between the vehicle’s drive shaft and axle speeds [8], an infrared detection system [9], and a device that generates a light pulse train whose amplitude changes when ice is present [10].There are also surface sensors meant to detect ice conditions that utilize sensing materials other than the pavement material, and/or are based on an indirect assessment of the potential for icing given existing conditions rather than detecting actual icy conditions.

In general, the systems described above typically contain complex and hard-to-maintain hardware and software components. Existing embedded systems involve utilization of sensing materials that are different from the surrounding material. In such cases, there is potential for a thermal island effect (i.e., a sensor having a different condition compared to the surrounding roadway surface). Furthermore, there is no distinction made between ice that may exist within the near-surface pores of the concrete material without surface ice (herein referred to as “frozen” condition) and the surface ice condition. High initial and long-term costs, complexity, and accuracy issues have prevented the wide-spread use of such systems on road networks nationwide.

The primary objective of this research program was to develop a new low-cost embedded sensor system for the detection of surface ice, water, frozen pavement, and dry conditions on roadways, bridges, and runways. The proposed methodology is based on near-surface and surface resistance measurements in the concrete sensing material as well as surface temperature measurements. To achieve the research objective, a set of laboratory tests was performed on two prototype sensors to verify the sensor’s effectiveness in detecting various environmental surface conditions (especially surface ice condition). A preliminary set of algorithms is proposed to determine the surface conditions based on the laboratory tests performed to date. 

Concrete was used as the sensor material in this initial development of the proposed ice sensor because surface ice can develop more rapidly on bridge decks compared to the approaching roadway, and most bridge decks in the U.S. have exposed concrete surfaces. Concrete pavements are also prevalent on U.S. roads and runways. It is envisioned that a similarly designed sensor can be developed for asphalt surfaces. However, this development was not within the scope of the study reported here.

The proposed intelligent sensor system is designed to offer the following advantages: ability to detect/distinguish a variety of surface conditions; relatively low cost; simple detection technology; suitability for mass production; and potential for a variety of wireless or wired hazard communications through local warning signals, web site information, transmission to autonomous vehicles, etc.

In this paper, a novel low-cost embedded sensor system is proposed that can detect surface ice, wet, dry, or frozen (ice in concrete pores) conditions on concrete roadways, runways, and bridge decks. This system utilizes measured changes in near-surface electrical resistance of concrete and the concrete surface temperature to determine surface conditions. Unlike other embedded sensors for ice detection, this sensor has a sensing material (concrete) that is compatible with the surrounding concrete and thus does not create a thermal island on the roadway or bridge deck.

## 2. Materials and Methods

### 2.1. Sensor Concept

The proposed sensor takes advantage of the fact that near-surface resistivity of concrete changes significantly when there is an ice layer or moisture at or near the surface. This sensor is designed to detect ice as it starts to form. The sensor could be designed to transmit warnings and other information including its location, surface temperature, and indications of black ice, frozen condition (ice in near surface concrete pores without surface ice), dry surface, or wet conditions.

The detection algorithm is based on measurements of electrical resistance of concrete (between embedded poles) and surface temperature measurements. The sensor is in the shape of a cylinder made with a concrete mix that is similar to those used in roadway pavements or bridge decks. It should be noted that the sensor is designed such that typical variations in concrete mix designs (concrete properties) could not mask the effects from ice or water conditions in the sensor output. 

Electrical resistance is measured between two sets of stainless steel poles embedded in concrete. Resistance measurements are made using two unbalanced Wheatstone bridge circuits (one for each set of poles). Two different types of poles are used:

“Look-Up-and-Side” (LUS) poles measure near-surface (within 3.2 mm of surface) electrical resistance changes in the concrete between the surfaces of the two poles in direct contact with concrete (“Sides”) as well as surface ice that may exist above the surface (“Up”).

“Look-Up” (LU) poles measure resistance changes directly above the surface of the concrete between the two poles. Under dry conditions, the resistance between LU poles is theoretically infinite as the space above the surface between LU poles could consist of air only.

The LUS pole was the only pole type used in the first prototype of the sensor. However, as discussed later, test results indicated the need for an additional type of pole (LU) in the sensor to reduce uncertainties under certain exposure conditions.

### 2.2. Sensor Geometry

**Figure 1:**
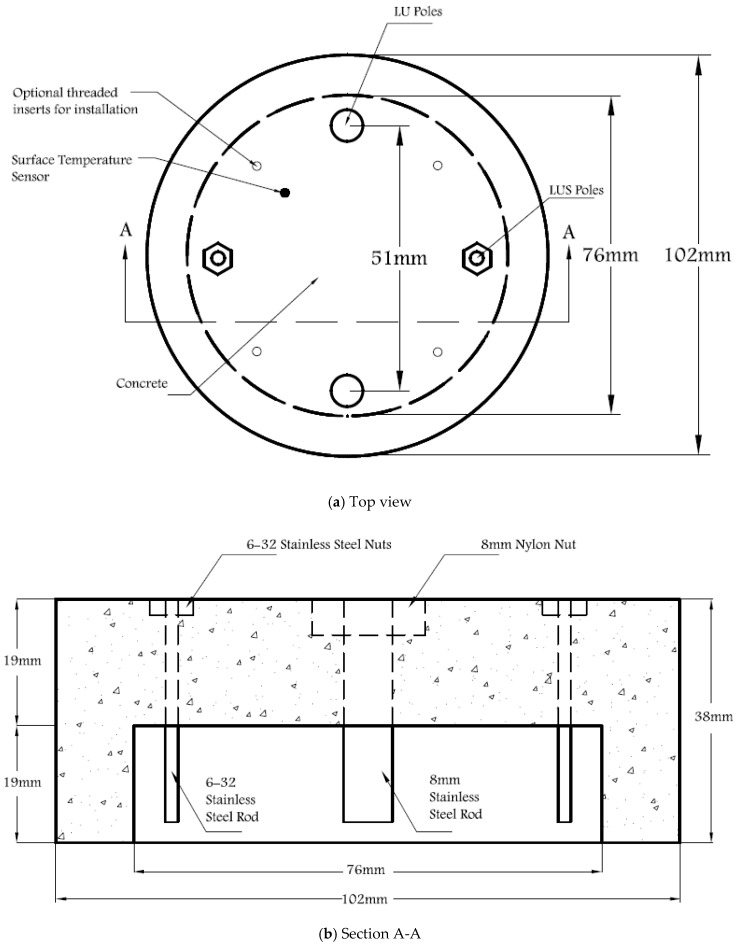
Dimensions of the proposed sensor: (**a**) Top view; (**b**) Section A-A.

**Figure 2:**
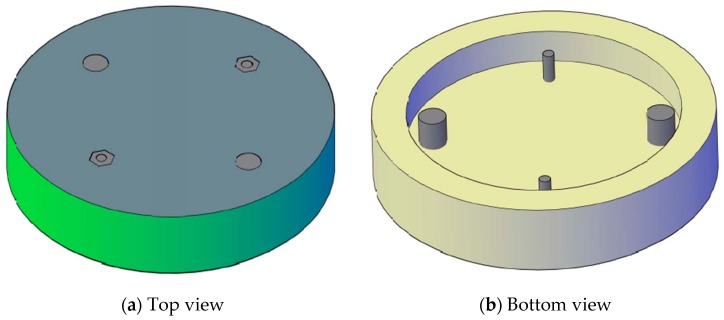
Schematic of the proposed sensor: (**a**) Top view; (**b**) Bottom view.

**Figure 3:**
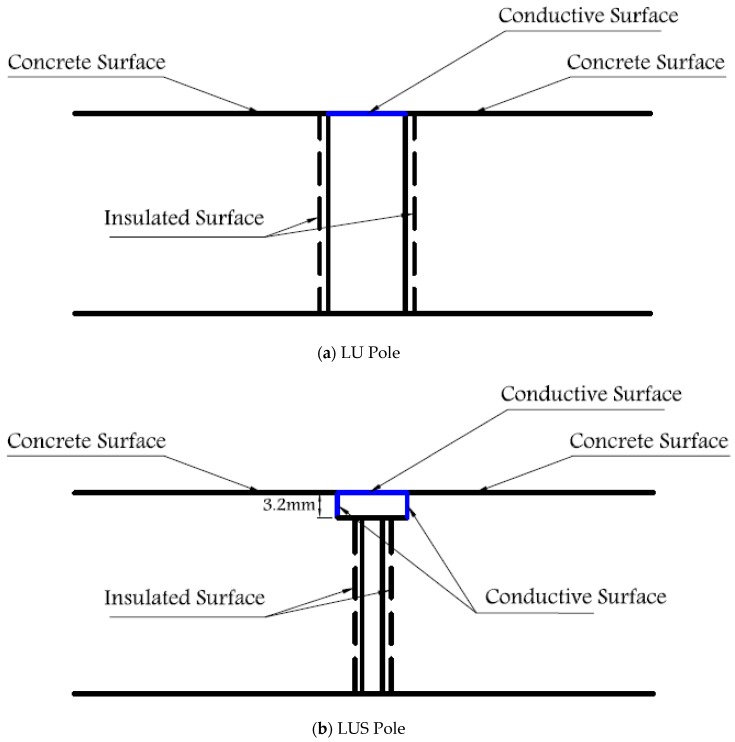
Pole details: (**a**) LU; (**b**) LUS.

The proposed cylinder-shaped concrete sensor has a diameter of 102 mm and a height of 38 mm and includes a 76-mm-diameter opening at the bottom of the sensor, as shown in Figure 1 and Figure 2. Two 6–32 stainless steel threaded rods with 6–32 stainless steel nuts are used as LUS poles (nuts and the end of rod are flush with the concrete surface), and two 8-mm-diameter stainless steel threaded rods are used as LU poles. The cross-sectional area of the 8 mm pole is nearly equal to that of the 6–32 rod and nut. This assures that both types of poles have the same conductive area projected upward (facing up) at the surface. All poles were electrically insulated with a layer of epoxy paint and two layers of electrical shrink tubing, where needed, along the embedded part of their length. The insulated and non-insulated areas of each pole type are shown in Figure 3. Furthermore, 8 mm plastic nuts were placed around the 8 mm rods at the surface to avoid any electrical contact between the concrete and the rods from the sides and to facilitate installation of the poles inside the mold prior to casting concrete.

The LU poles are included to address the ice formation on the top of their cross-sectional area only, since they are electrically exposed from the top only (i.e., they are sensitive to resistance changes above the surface of the sensor only). On the other hand, LUS poles are electrically exposed to changes in the near-surface concrete resistance as well as changes above the surface as shown in Figure 3. The use of LU poles allows a more definitive determination of surface ice, by helping to distinguish it from the frozen condition. LUS results alone may not be conclusive under some circumstances in determining whether the detected ice is at/above the surface or within the pores of concrete. This distinction is important since, as indicated later in this paper, there is a significant difference in surface friction (safety risk) between surface ice and frozen conditions.

**Figure 4:**
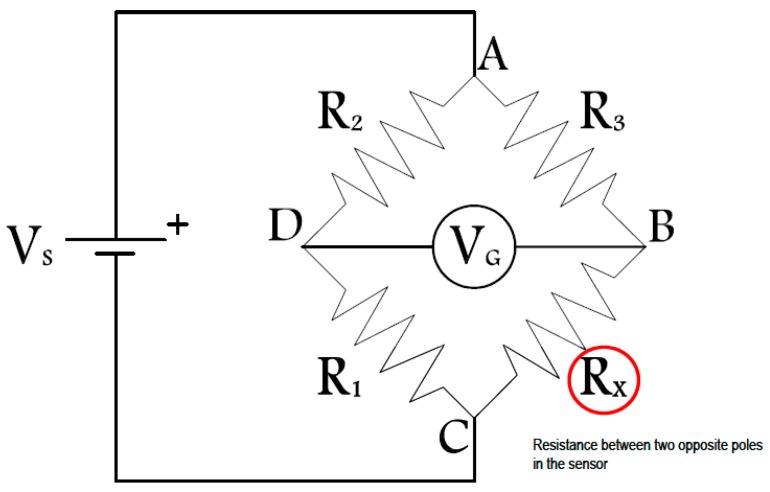
Wheatstone bridge.

In addition to serving as resistance measurement poles, the stainless-steel rods are also used for making electrical connections to the circuit board (instead of wires). This is done to eliminate the potential for long-term corrosion of conventional wires. The circuit board and other electronic components are housed within the sensor. They include two separate Wheatstone bridges (only one is shown in Figure 4), a power supply unit (battery), a wireless transmission unit (if applicable), a logic controller, and a temperature sensor. The sensing element for the temperature sensor is at the top surface of the concrete sensor.

The Wheatstone bridge has a low current demand. The demand can increase with the addition of wireless communications or other systems. The controller can be programmed to turn on the ice monitoring only when the temperature is low, eliminating the need for continuous monitoring. Another powering option may involve small solar cells placed on the top surface outside of the pole grid area. The battery could be in a cavity accessible from the top. Considering other commercial wireless products, a battery life of 5–10 years may be achievable, pending field evaluations and testing. 

The controller determines the surface condition based on inputs from the two Wheatstone bridges as well as the surface temperature, in accordance with the developed decision algorithm. The outcome can then be transmitted in a variety of wired or wireless modes. The inside surface of the cavity that contains the electronic components must be sealed (coated with epoxy and/or provided with a liner) to avoid moisture penetration. A cover is used to seal the opening and prevent possible damage to the electrical circuits.

### 2.3. Sensor Installation

The proposed sensor can be used in new constructions or retrofitted on existing roads or bridge decks. The installation (embedment) of this sensor in existing concrete pavements or bridge decks involve using a 102-mm-diameter core drill bit to remove a core and replacing it with the concrete sensor. This is a common diameter for cylinders used in quality control tests to determine the concrete’s compressive strength. Coring machines with this bit size are widely available and accessible to concrete contractors, thus making the field installation relatively easy. A portland-cement-based mortar (regular or fast-setting) should be used to bond the concrete sensor to the existing concrete pavement or bridge deck to avoid any thermal incompatibility or thermal island effect.

**Figure 5:**
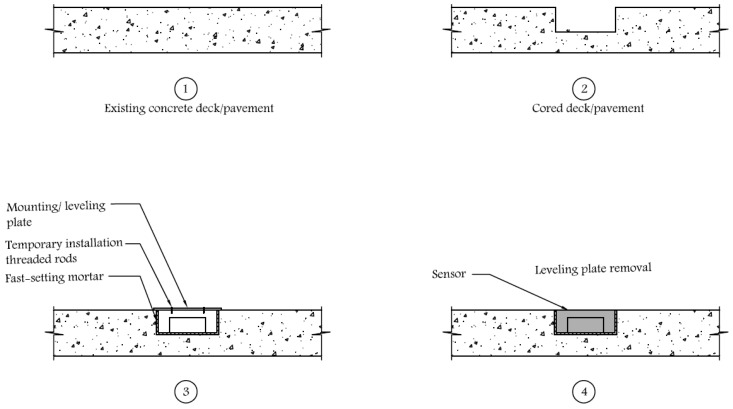
Installation of sensor on existing concrete deck/pavement.

There are several options available to the installer to provide a flat transition between the existing surface and the sensor. One such option involves the temporary attachment of a mounting (or leveling) plate on the top surface of the sensor during installation and hardening of the cement mortar (Figure 5). The mounting plate has a slightly larger diameter than the sensor, such that it could rest on the edges of the cored opening in the concrete. The cement mortar covers both the sides and the bottom of the sensor and completely fills the gap between the bottom of the sensor and the bottom of the cored hole. After the cement mortar is set, the mounting plate is removed by first removing the screws holding it to the top surface of the sensor. The sensor can be removed using the same type of coring device used for installation.

### 2.4. Concrete Mix

The concrete mix used to make the sensor prototypes consisted of the following: 15.6% (by weight of dry materials) Portland cement (Type I), 31.2% sand, 46.9% pea gravel, and 6.3% water. The water/cement ratio was 0.4, as specified by the Wisconsin Department of Transportation’s standard specifications for concrete Grade A. No other admixtures or additives were used. The specimens were stripped from their plastic mold two days after casting of the concrete and were then wet cured in the laboratory for 7 days. 

### 2.5. Sensor Prototype I (SP-I)

SP-I was the first sensor prototype used in this research. SP-I was similar to the proposed sensor described earlier except that all four poles used were LUS poles (i.e., no LU poles were used). Additionally, the diameter of the opening on the bottom of the sensor was slightly smaller than the proposed sensor. 

Four stainless steel nuts were embedded in the sensor mold prior to casting of the concrete. These poles were flush with the top surface of the sensor. Electrical wires were placed in the nuts before tightening the threaded rods. Unlike the proposed sensor that incorporates stainless steel poles for electrical connections, common electrical wires were used to connect the nuts (poles) to the Wheatstone bridge in the SP-I prototype.

### 2.6. Sensor Prototype II (SP-II)

This sensor prototype had the same external dimensions as the SP-I with a 76-mm-diameter opening at the bottom. Two 6–32 stainless steel threaded rods with 6–32 stainless steel nuts were used as LUS poles similar to those used in SP-I. Two 8-mm-diameter stainless steel threaded rods were used as LU poles. The LU poles were included to better distinguish the frozen condition from surface ice, especially when the surface is contaminated with deicing chlorides and rubber shavings. All poles were insulated with a layer of epoxy paint and two layers of electrical shrink tubing throughout the embedded part of their length. This was meant to electrically isolate the LU poles from the concrete throughout the exterior surface of the pole (i.e., the only conductive surface was the cross section of the pole at the surface of the sensor).

In SP-II, stainless steel rods were used in lieu of wires that were used in SP-I. Stainless steel rods were chosen to address the long-term corrosion problem caused by exposure to moisture.

### 2.7. Electrical Circuit

As discussed earlier, the output of an unbalanced Wheatstone bridge was used in this research as an indicator of resistance changes between two opposite poles in each sensor. The resistance between the two sensor poles was included as a leg of the Wheatstone bridge shown in Figure 4.

The Wheatstone bridge has been used to measure resistance changes in a wide variety of different applications including load cells and strain gages. It offers a passive low-cost option to assess fluctuations in electrical resistance. 

In a Wheatstone bridge, there are four legs, each consisting of a resistor. Three of these were constant precision resistors (R_1_, R_2_, and R_3_). R_x_ was the resistance of concrete being determined. The output voltage (V_G_) of an unbalanced Wheatstone bridge can be calculated using the following equation:

(1) $$V_{G}=\left(\frac{R_{x}}{R_{3}+R_{x}}-\frac{R_{2}}{R_{1}+R_{2}}\right)\times V_{s}$$

The excitation voltage (Vs) and resistors on the three known legs of the Wheatstone bridge (R_1_ thru R_3_) used in the prototypes were as follows: V_s_ = 6 VDC; R_1_ = 150 kΩ; R_2_ = 100 kΩ; R_3_ = 10 MΩ. This choice of resistors was made after many trials to achieve a desired wide range of output for various conditions, in which high-resolution voltage measurements were not needed. With this choice of resistors and an excitation voltage of 6 V, the lowest and highest sensor output voltages ranged from approximately −2 (for fully wet conditions) to under +2 V for fully dry conditions.

### 2.8. Friction under Various Surface Conditions

To fully benefit from the knowledge about surface conditions, it is important to determine and understand friction levels under different surface conditions. This is important because sensor outputs could then be quantitatively associated with safety hazards.

A device called “British Pendulum Tester” (based on the U.S. test standard ASTM E303 [11]) was used for measuring friction. In this test, a pendulum is released from a pre-determined height. The rubber pendulum slider contacts (slides against) the surface and rises again. The loss of energy due to friction results in a difference between the height of the slider before and after meeting the surface. The result is a measure of friction and is reported in BPN (British Pendulum Number).

**Figure 6:**
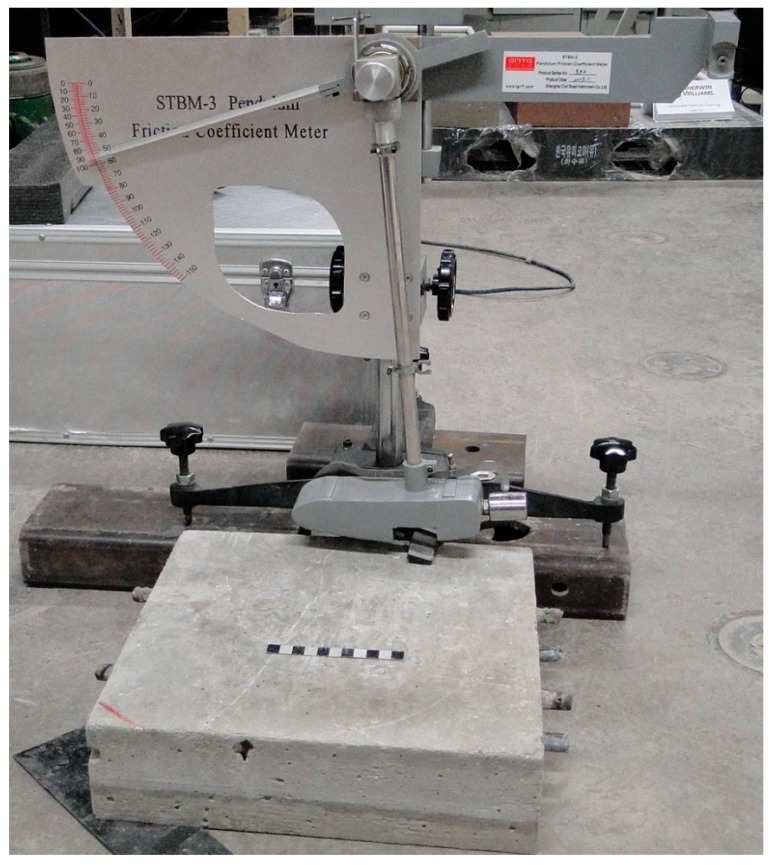
British Pendulum Tester and the concrete slab specimen.

Tests on five different surface conditions were conducted. These include dry surface, wet surface, icy surface (black ice), frozen surface (i.e., frozen water in the concrete pores that are near the surface), and frozen-icy surface (combination of frozen and black ice conditions). A 457 mm by 457 mm concrete slab surface was used for this test as shown in Figure 6. The concrete slab was first tested under dry conditions to obtain the baseline BPN. The surface was then sprayed with water to achieve a wet surface condition, and the friction test was performed again. The specimen was then moved into a freezer (−20 °C) and left there for approximately 24 h. The specimen was removed from the freezer and water was sprayed on the top surface to form surface ice. The specimen was then tested to find the BPN under surface ice condition.

For the frozen test, the slab was placed face down in a water-filled tray for 24 h. The specimen surface was then wiped to remove free water, and was then placed in the freezer for another 24 h. The concrete specimen was tested again to determine the BPN under frozen conditions. Finally, the process for the frozen condition was repeated, but this time water was sprayed on the surface of the concrete slab, and a frozen-surface ice condition was created for testing. Six readings were taken for each condition, and an average value was calculated.

**Figure 7:**
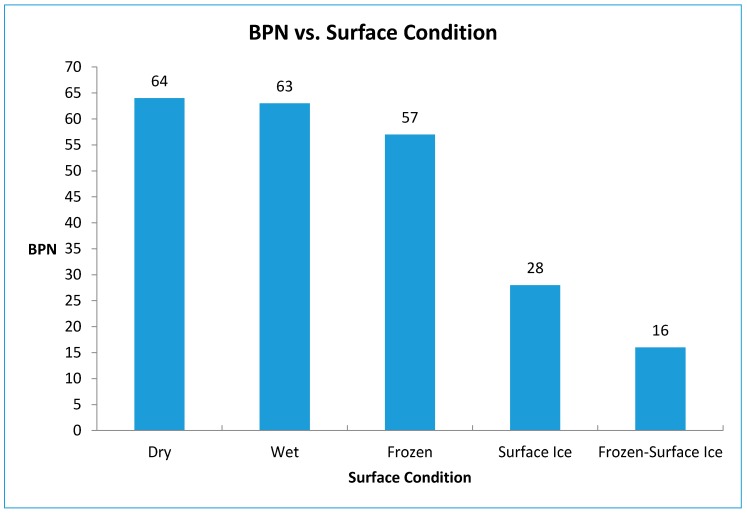
Friction results under different surface conditions.

Figure 7 shows the relationship between surface condition and the BPN. As expected, BPN decreases as ice forms on the surface. The lowest friction occurs when frozen water exists in the concrete pores near the surface together with a layer of surface ice. In this case, BPN drops from 57 for frozen concrete (without surface ice) to 16 when there is ice on the surface of frozen slab. A significant reduction in the BPN resulted from the combination of the presence of frozen water particles in the pores and the icy surface.

Results indicate that the presence of ice inside concrete pores without surface ice poses a far less safety risk than the surface ice condition. This demonstrates the importance of distinguishing the two forms of ice in the sensing system.

### 2.9. Prototype Testing and Verification

The development and verification aspect of this work involved building and testing two sensor prototypes (SP-I and SP-II). These two prototypes were used to generate test data for the development of the preliminary decision algorithm and to establish the feasibility and effectiveness of the proposed sensor concept through laboratory testing. A Wheatstone bridge circuit built on breadboard was assembled for testing purpose. Using Equation (1) and knowing the values of R_1_, R_2_, R_3_, and V_G_, the values of R_x_ could be calculated. 

To understand and determine the possible range of resistance, tests were performed under different environmental conditions. In all tests described below, the sensor output was monitored every few minutes as the surface temperature increased from below freezing temperatures up to room temperature.

In the laboratory experiments, the surface temperature was monitored using an infrared non-contact temperature sensor even though the actual sensor in a field installation would have an embedded temperature sensor at the surface. This was done so that multiple measurements could be made at different locations and averaged. The recorded temperature was the average of 4–6 readings at different spots on the surface of the sensor. The temperature was measured along with V_G_. The following environmental conditions were tested:

#### 2.9.1. Dry Condition (DR)

For DR tests, the sensor was first placed in an oven to be dried for approximately 24 h at a temperature of 48 °C. After that, the sensor was taken out of the oven and allowed to cool down. Subsequently, it was placed in the freezer for 24 h at a temperature of −20 °C. The sensor was taken out of the freezer and connected to the Wheatstone bridge to monitor the output of the sensor. 

#### 2.9.2. Surface Ice (Black Ice) (SI)

**Figure 8:**
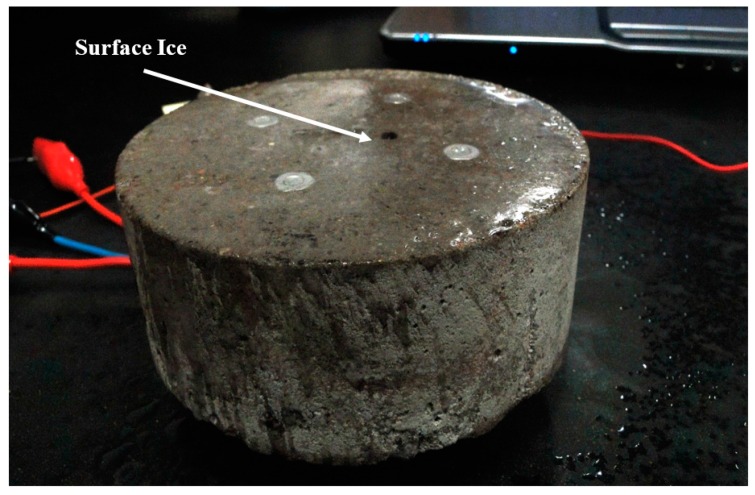
Surface ice formation on cold SP-I sensor.

For the SP-I prototype (with LUS Poles), the dry sensor was placed in a freezer (−20 °C) overnight. Just prior to the beginning of testing, the sensor was removed from the freezer and sprayed with room-temperature water from a spray bottle. The surface ice was then formed (Figure 8), and measurements were periodically taken as surface temperature increased up to room temperature.

For the SP-II prototype (with LU Poles), a similar procedure was used for the SI tests. The sensor was dried out by placing it in the oven with temperature of 35 °C for 24 h. The sensor was then put in the freezer for another 24 h. While still cold, water was sprayed on its surface to form surface ice. The LU poles were connected to the electrical circuit to monitor the changes in the resistance associated with the changes in the temperature.

#### 2.9.3. Frozen (without Surface Ice) (FR)

For tests on the SP-I prototype (LUS poles), the top of the sensor was immersed in water for a few hours to allow its near-surface pores to be filled with water. Subsequently, the sensor was removed from water and the excess surface water was wiped off. The sensor was then placed in the freezer (temperature of −20 °C) overnight to allow water in the pores to freeze. The sensor was removed from the freezer just before testing. Unlike the SI test, water was not sprayed on the surface. This condition represents the situation in which the pavement is saturated before freezing without formation of surface ice. Temperature and sensor measurements were taken until the temperature of sensor reached the room temperature. It should be noted that the FR test would also provide results under wet conditions. As the temperature increases, the ice in the pores changes to water.

For tests on the SP-II prototype (LU poles), a slightly different procedure was used. The LU poles were first covered with electrical tape to avoid contact with moisture. Water was sprayed on the surface several times for about an hour to make sure the pores were filled with water (without standing water). The sensor was then put into the freezer for 24 h, and then moved back to the lab to monitor the change in output voltages as temperature changed. 

#### 2.9.4. Frozen Concrete with Surface Ice (FR-SI)

This condition is a combination of the SI and FR conditions described above. The FR procedures were followed, but, after removal from the freezer, room-temperature water was sprayed onto the top surface to form a surface ice layer on top of the frozen surface.

#### 2.9.5. Additional Tests (SI-SW, FR-CC, SI-CC, SI-RC, CI, and Ice)

There were several other tests performed on the SP-I prototype to address the potential of surface contamination due to deicing salt or rubber residue from tires. Finally, a test involving the placement of crushed ice on the sensor was performed to simulate the effects of unbonded ice (not fully adhered to concrete) and snow. Finally, the resistance of a thin ice layer (without concrete) was also measured.

##### Surface Ice with Saltwater (SI-SW)

In this test, the sensor was placed in the freezer for about 24 h. Subsequently, it was moved to the laboratory and its surface was sprayed with water to form surface ice. The test was started and readings were taking until the temperature increased up to −4 °C, then saltwater (SW) was sprayed on the sensor, and readings were taken as the temperature increased. The saltwater was a 6% NaCl solution at room temperature.

##### Frozen with Chloride Contamination (FR-CC)

The SP-I prototype was submerged in water for about 2–3 h, and then placed in the freezer for 24 h. The sensor was already contaminated with salt due to the previous test. Readings were taken after the sensor was removed from the freezer and connected to the Wheatstone bridge. Readings were taken as the temperature increased from −15 to −10.5 °C, after which saltwater was sprayed on the surface.

##### Surface Ice on Chloride Contaminated Surface (SI-CC)

In the previous test, saltwater was sprayed onto the sensor, and that caused the sensor to be contaminated with chlorides. As sodium chloride penetrates the concrete pores, the output voltage and resistance are reduced. In this test, a condition when the sensor is previously contaminated with chlorides was simulated. The dry sensor was placed for 24 h in the freezer with a temperature of −20 °C. The sensor was then moved to the laboratory and was connected to the Wheatstone bridge circuit. Water was sprayed on its surface to create an icy surface. Two tests were done using this approach.

##### Surface Ice on Rubber Contaminated Surface (SI-RC)

Rubber residue from passage of tires can be present on the roadway surface. In this test, an attempt was made to simulate the condition where there is contamination due to rubber residue on the surface of the sensor. Powdered rubber (from an unrelated wear test on concrete) was rubbed on the surface of the sensor prior to this test. This sensor was previously contaminated with chlorides as well. The SI procedures were then followed.

##### Crushed Ice (CI)

The SP-I prototype was placed inside in the freezer (−20 °C) for 24 h. While it was in the freezer, crushed ice was placed on the top surface of the sensor. The sensor was then moved to the laboratory inside an insulation box to slow the temperature increase. The sensor output voltage was monitored as the temperature increased and ice melted.

##### Ice Test (without Concrete)

To better understand the electrical properties of the ice at different temperatures, tests were performed on an ice layer without concrete. A plastic container, which had served as a mold for casting of concrete sensors, was filled with 1.6 mm (1/16 in) of water. Stainless steel poles were placed in the same arrangement as those used in the sensor. 

## 3. Results

### 3.1. Dry Test (DR) 

**Figure 9:**
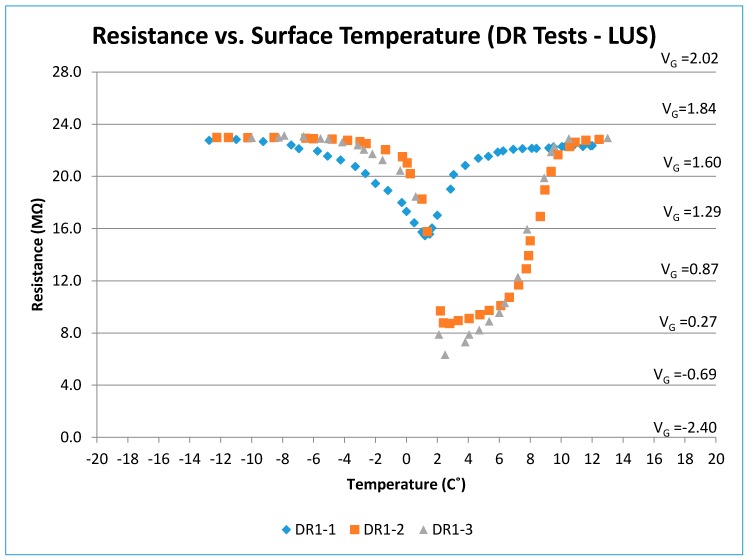
Resistance (and voltage) versus surface temperature for all DR tests (LUS Poles).

Three DR tests were performed on the SP-I sensor (DRI-1, 2, and 3). The sensor was dried in the oven for 24 h prior to DRI-1. In tests DRI-2 and DRI-3, the sensor was not dried in the oven before testing. The sensor was then placed in the freezer for 24 h. Figure 9 shows resistance and voltage changes with temperature for the all DR tests on the SP-I prototype. As the surface temperature approached and exceeded 0 °C, condensation formed on the surface, which caused a dip in the output voltage and resistance. However, aside from the condensation effects, the output voltage (for V_S_ = 6 V) was on the order of 1.75 V (or resistance of 22–23 MΩ).

In the DRI-2 test, the condensation affect was larger (degree of condensation was related to the humidity of the laboratory). The dry resistance was again on the order of 22–23 MΩ. The DRI-3 test did not differ significantly from DRI-2, except for a more pronounced condensation effect. 

It should be noted that, at cold temperatures below −8 °C and at warmer temperatures above +8 °C, the voltage output for the three DR tests were similar and approximately equal to 1.75 V. The DRI-1 results began to diverge from DRI-2 and DRI-3 within a temperature range from −6 to +10 °C. The presence of moisture due to condensation drives the output voltage and resistance to lower levels. As the moisture disappears at higher temperatures, the output voltage and resistance values for all DRI tests converge again.

### 3.2. Frozen Test (FR)

**Figure 10:**
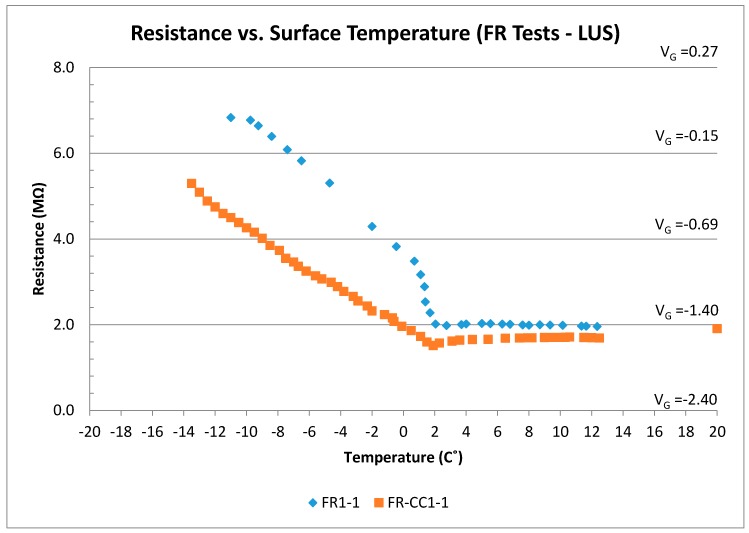
Resistance (and voltage) versus surface temperature for all FR tests (LUS Poles).

This test was conducted to understand the effect of the presence of frozen water in the concrete pores and to see how the change from pore ice to pore water would affect the electrical resistance between poles. The voltage and resistance results are shown in Figure 10. Within the temperature range from −11 to 2 °C, the voltage (and resistance) decreased as the temperature increased. The output dropped rapidly as ice within the concrete pores began to melt. There is a nearly linear relationship between voltage (resistance) and temperature at temperatures below freezing point. The voltage levels under the FR condition are much lower than the DR results described earlier. For temperatures above 2 °C, voltage and resistance values stayed nearly constant. This can be explained by the fact that the ice in the concrete pores converted from solid (ice) into liquid form, and the output observed was associated with a fully moist concrete condition.

The resistance of concrete is related to the presence of moisture in the pore structure. Therefore, the measured resistance and voltage both decrease as water content increases. Voltage output for frozen concrete ranged between +0.1 and −0.8 V (7.0–3.5 MΩ), while moist concrete output voltage was on the order of −1.4 V (2.0 MΩ). The wide gap in measured voltage and resistance values from dry to wet conditions indicates that typical variations in concrete properties may not be sufficient to mask the effects from ice or water. Spragg et al. [12] report coefficients of variance of 4.36% and 13.22% for bulk resistivity of concrete when tested at the same laboratory and multiple laboratories, respectively. The relative insensitivity of the outcome to dry concrete resistance variations can be illustrated by varying the measured dry concrete resistance (about 23 MΩ) by a reasonable amount (say ± 25%) and plugging the result into Equation 1. The output voltage would still be outside the range of outputs from ice or water conditions observed when dry R_x_ was 23 MΩ.

**Figure 11:**
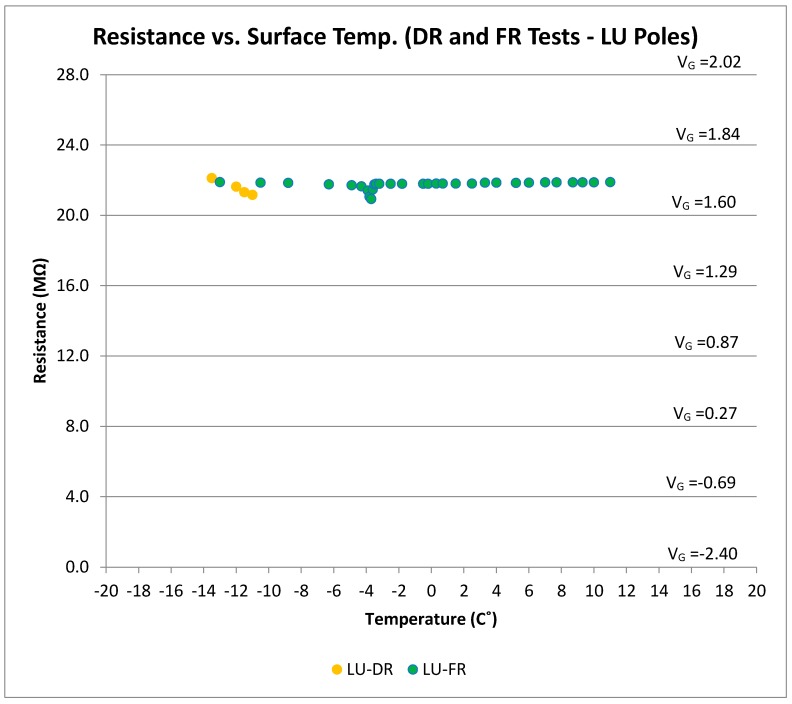
Resistance (and voltage) vs. surface temperature for DR and FR tests (LU Poles).

Figure 11 shows the output voltage and resistance of the SP-II prototype (LU poles) under frozen condition. It can be observed that the output voltage of the sensor was essentially constant and not a function of temperature. As expected, the LU poles were insensitive to the frozen, or the subsequent wet, condition of the concrete material between the two LU poles. Thus, LU poles will only be responsive to either surface ice condition, or wet conditions that cover the top surface of the pole with water (standing water). This would overcome any uncertainties associated with distinguishing surface ice and frozen conditions in LUS poles discussed later. 

### 3.3. Surface Ice Test (SI)

Surface ice is one of the most dangerous pavement condition as indicated in the friction tests reported earlier. The focus of interest in this research, therefore, was on proper detection of surface ice conditions.

**Figure 12:**
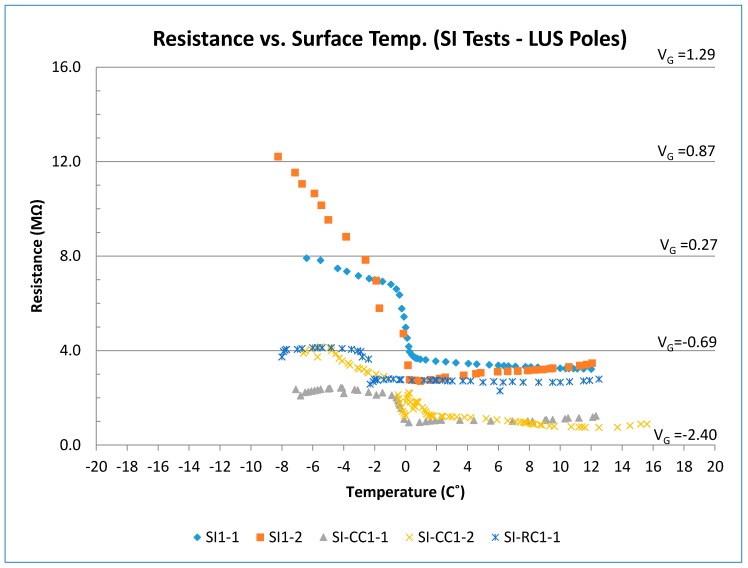
Resistance (and voltage) versus surface temperature for all SI tests (LUS Poles).

The voltage (resistance) and surface temperature readings for the SI-I tests (LUS poles) are shown in Figure 12. There was a generally linear relationship between output voltage and surface temperature when there was surface ice present, and the output voltage was on the order of 0–0.25 V. As the ice melted, the output voltage dropped to −0.75 V. The output voltage under moist condition was in the range from −0.75 to −1.0 V.

**Figure 13:**
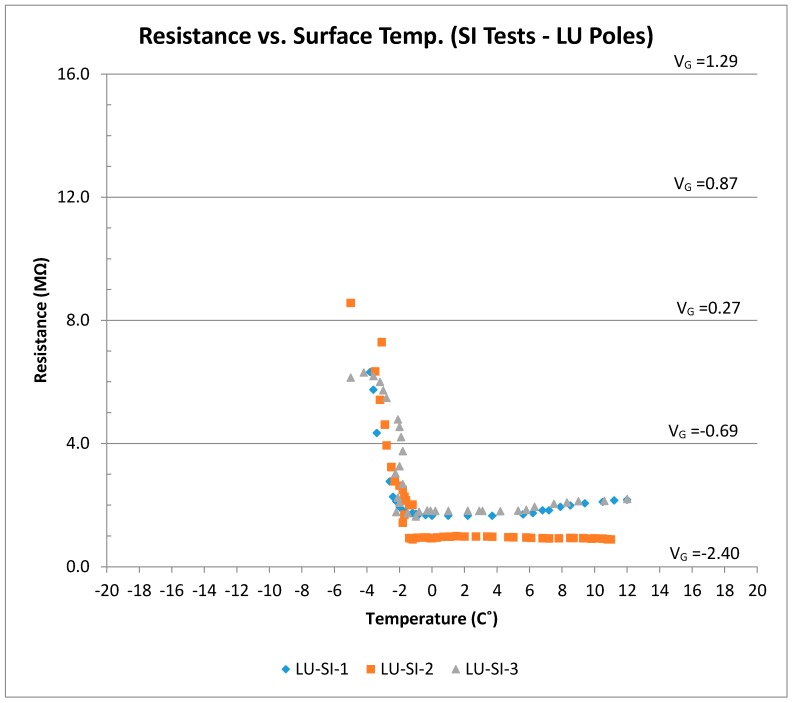
Resistance (and voltage) versus surface temperature for all SI tests (LU Poles).

In the first test on the SP-II prototype (LU poles), the output voltage and resistance was monitored as the surface temperature of the sensor increased from −11.5 to 14 °C. The first four readings were taken while the sensor was cold and dry without moisture on its surface. Water was then sprayed on the surface and surface ice started to form (Figure 13). Ice formation on the surface caused a rapid decrease in the output voltage and resistance values. The change continued as the ice was melting at temperatures around 0 °C. Voltage and resistance was nearly stable as the temperature changed from 0 to 12 °C.

### 3.4. Other Tests

**Figure 14:**
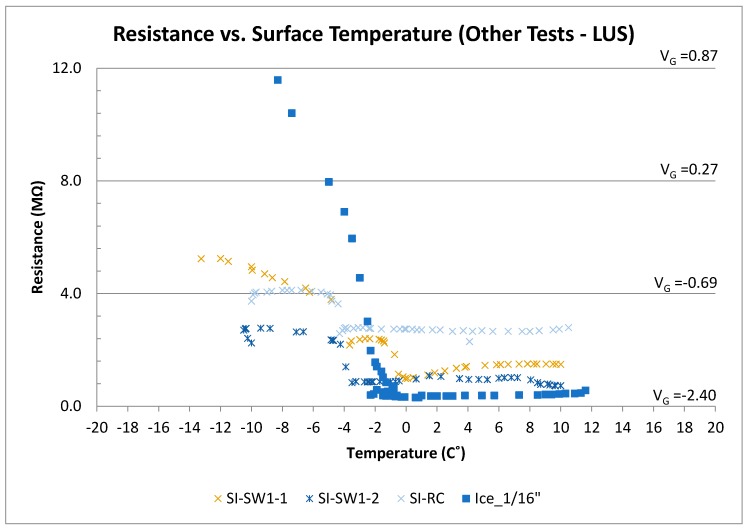
Resistance (and voltage) versus surface temperature for other tests (LUS Poles).

Figure 14 shows results of the other tests performed using the LUS poles (SP-I prototype). In the SI-SW tests, for the range of temperature between −12 and −4 °C, the plot for SI-SW I-1 showed an expected trend for surface ice. The initial increase in voltage was due to the icing of water as it was sprayed on the surface. After saltwater was sprayed, temperature increased rapidly, and output voltage and resistance decreased. The presence of salt reduced the electrical resistance of concrete. For temperatures between −2 and 2 °C, the SI-SW I-1 test showed a gradual reduction in output voltage and resistance. 

The SI-SW I-2 test was performed on the same sensor following conclusion of the SI-SW I-1 test. Therefore, it is thought that the deicing salt used in the first test was absorbed by the concrete and stayed in its pores. This caused the sensor to be contaminated with salt, which caused a difference between results of SI-SW I-1 and SI-SW I-2. For the temperature above 2 °C, the SI-SW I-1 test showed the normal trend of concrete having melted ice on its surface. Test SI-SW I-2, however, shows the effect of salt contamination within the concrete pores and on the surface where voltage and resistance were lower prior to saltwater spray.

In the FR-CC test, there was a rapid increase in temperature and a rapid decrease in output voltage because of the addition of saltwater. At temperatures above 0 °C, the output voltage was on the order of −2.0 V, and the resistance was on the order of less than 1.0 MΩ. The lower resistance under wet conditions is due to chloride contamination. The presence of salt affects the conductivity of concrete and the overall resistance decreases. However, there is still a significant difference in output voltage between surface ice and wet condition under chloride contamination. This allows the sensor to work even in chloride-contaminated environments. Nevertheless, the presence of salt affects the output, and this is considered in the preliminary decision algorithm proposed for the sensor.

It is thought that the difference in the results of the two tests may be due to the amount of chloride contamination of the sensor in the two tests. As the ice fully melts, and temperature increases up to the room temperature, resistance and voltage results for both tests (SI-CC I-1 and SI-CC I-2) fall within a range from −1.75 to −2.0 V (≈1 MΩ).

In the SI-RC tests, since the sensor was already contaminated with salt from previous tests, the response was like the chloride-contaminated tests described earlier. Overall, the rubber shavings on the sensor surface did not appear to change the results drastically. Outputs stayed within the range of icy surface results in clean or chloride-contaminated tests.

In the crushed ice test, as surface temperature increased (crushed ice melted), the output voltage and resistance decreased. At lower temperature, the output voltage level was similar to the dry condition even though there was crushed ice on the surface. It is believed that, due to the voids between the crushed ice/snow particles (ice not fully adhered to concrete), the electrical resistance will not be similar to that under the surface ice condition. In fact, the output voltage was close to the that of the dry condition when the crushed ice had not melted. This pattern could be seen in the temperature range from −10 to −3 °C. As temperature increased and ice converted into liquid form, water became ice again because of the cold sensor. The output voltage reached −0.94 V (resistance ≈ 3.0 MΩ) for temperatures above 1 °C.

Tests on the ice layer alone (without concrete) showed that the ice resistance has a linear relationship with temperature. This was seen in surface ice tests on the sensor as well. As the thickness of ice increases, the output voltage decreases.

## 4. Discussion

### 4.1. Discussion of SP-I Results (LUS Poles) 

**Figure 15:**
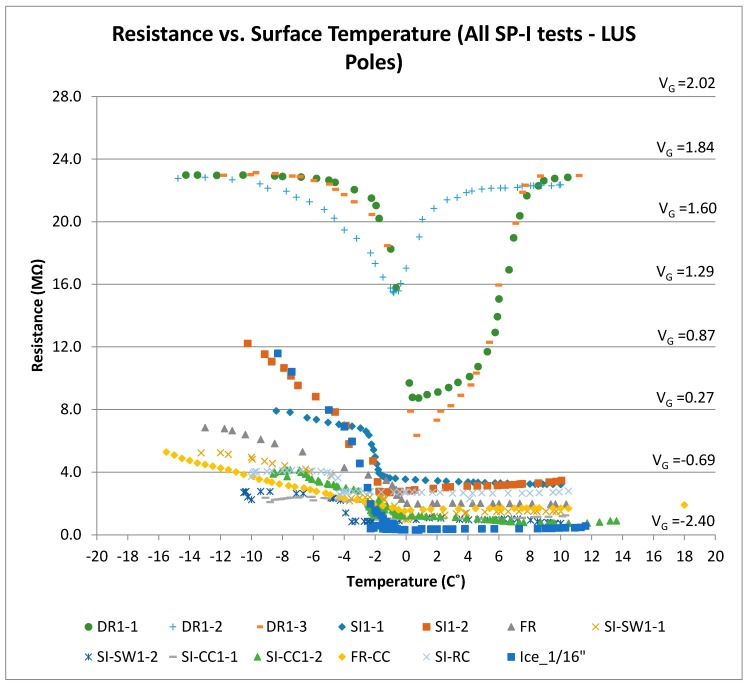
Resistance (and voltage) versus surface temperature for all SP-I prototype tests (LUS Poles).

Figure 15 shows the results of all LUS pole tests together. It should be noted that the results of the cold dry sensor (for a temperature or range of temperatures) are well separated from the results for surface ice conditions. Output as voltage or resistance could be used to indicate surface condition. For instance, for the temperature range from −8 to −2 °C, a dry condition determination could be made if the output voltage were between +1.75 and +1.25 V. On the other hand, if the voltage were between +1.0 and 0 V, a surface ice determination could be made. The output voltage response is linear with temperature in this zone, and the slope is on the order of −0.1 V/°C. 

The presence of chlorides did not affect the overall shape of the voltage–temperature response. However, it drove the resistance and voltage values lower. Therefore, a range of voltage or resistance should be found for the sensor’s output that would include the effect of salt presence and the surface condition that is associated with it (dry, frozen, surface ice, etc.). There was also no interference between the conditions of interest (surface ice, frozen, frozen-surface ice) and the dry condition under the same temperature or range of temperatures. There was, however, one possible interference area between surface ice and frozen conditions when chloride contamination was considered. Since tests indicated that friction values for the frozen surface was only slightly lower than those under the dry condition (and much higher than those under the surface ice condition), the frozen condition should be clearly distinguished from surface ice or frozen-surface ice conditions. Friction for the frozen-surface ice combined was the worst (lowest), so the decision algorithm should be designed to fully separate various conditions. Since the LUS pole results could fall within the zone of interference for salt-contaminated surfaces, the LU poles were developed to conclusively separate the two conditions.

When surface temperature is above −2 °C and the output voltage is within a range from −2.25 to −0.75 V, the surface condition is wet. As the output voltage approaches −2.25 V, the wetness increases.

### 4.2. Discussion of SP-II Results (LU Poles) 

**Figure 16:**
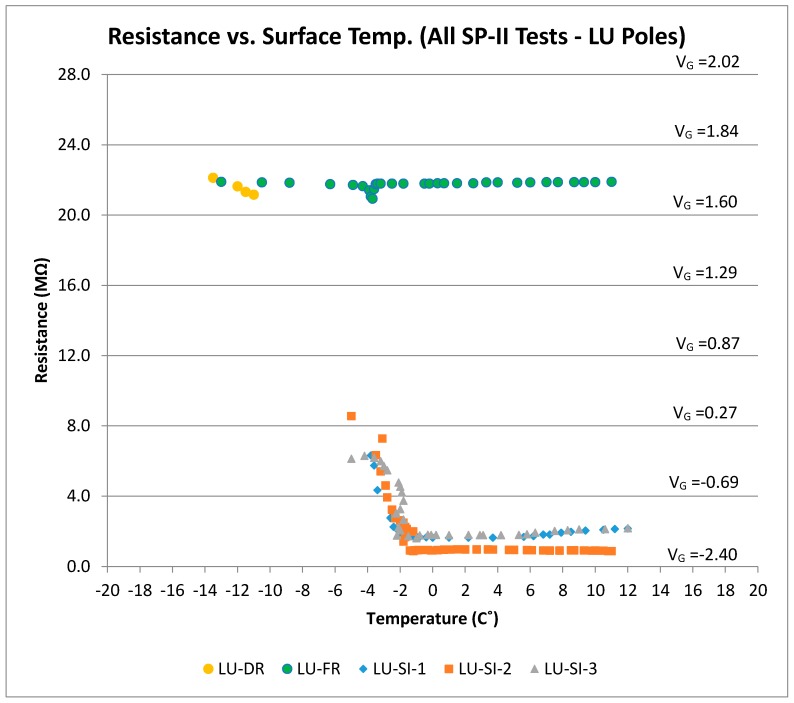
Resistance (and voltage) versus surface temperature for all SP-II prototype tests (LU Poles).

Figure 16 shows all results for the LU poles under surface ice and frozen conditions. The output voltage for LU poles under the frozen condition was similar to the cold dry results. Therefore, the frozen condition could be successfully distinguished from the surface ice condition using the LU pole results. With the addition of the LU poles, there is no longer any ambiguity between the two outcomes. Algorithms could thus be developed based on these results such that the most severe warning could be issued only when there is confidence about the presence of surface ice. The frozen condition, however, could be detected by the LUS poles, and appropriate and less severe warning can be issued. As determined from the friction tests, the worst condition (lowest friction) occurs when there is frozen concrete and surface ice combined.

### 4.3. Preliminary Decision Algorithm

**Figure 17:**
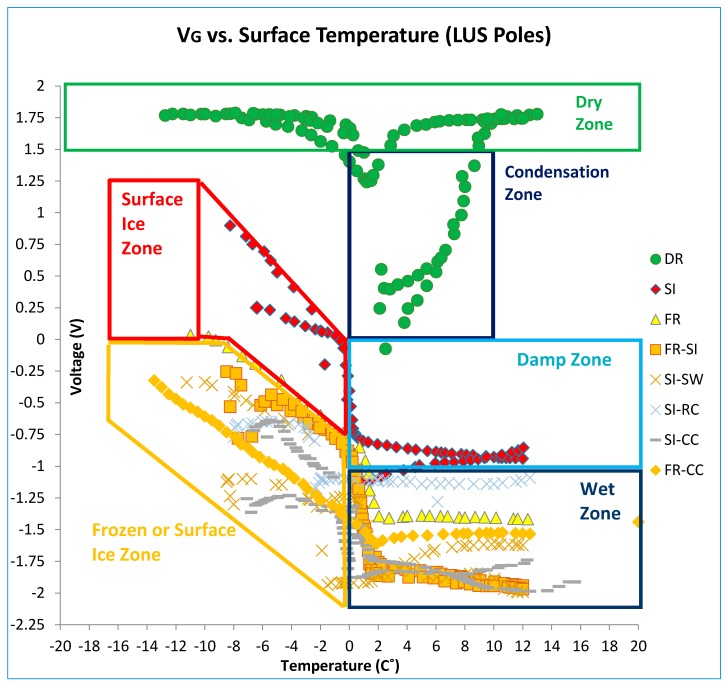
Zones of surface condition based on LUS laboratory test results.

**Figure 18:**
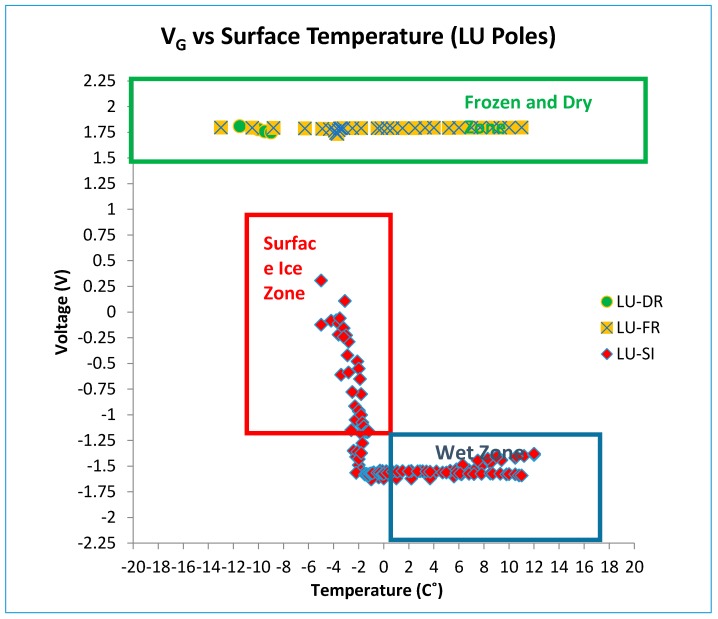
Zones of surface condition based on LU laboratory test results.

**Figure 19:**
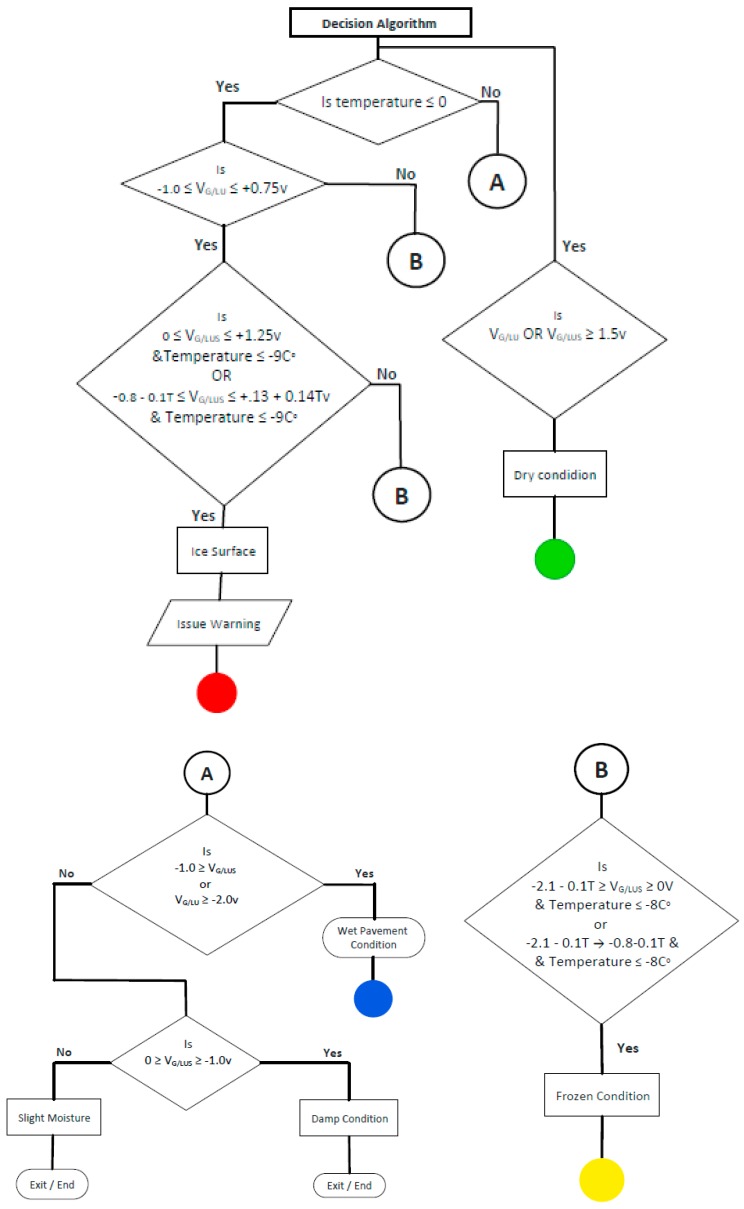
Preliminary decision algorithm for the concrete sensor.

Figure 17 and Figure 18 illustrate the various condition zones (surface ice, frozen, dry, and wet) that are currently assigned for LUS and LU poles, respectively, based on these laboratory results. Table 1 shows these zones in tabular form. Using these zones, a preliminary decision algorithm has been developed. The algorithm (Figure 19) does not address all possible combinations of voltage and temperature. However, it addresses those directly associated with the experiments performed to date. The algorithm should be further refined through controlled field testing.

The proposed sensor system can be further developed through field testing and enhancement of the detection algorithm. In this study, the sensor output was related to surface and near-surface conditions (ice, water, etc.). In the future, the sensor output could potentially be used for determining surface friction as a direct measure of safety risk. Durability assessments should also be made, both in terms of long-term concrete wear and freeze–thaw scaling, and with respect to battery life. Various communication and transmission systems including those intended for local warning, for traffic operations centers, and for autonomous vehicles could be developed in the future. A similar sensor system for asphalt pavements should also be considered in future sensor development work. 

## 5. Summary and Conclusions

1. This research aimed to develop a novel sensor system for detection of ice and water conditions on roadways, runways, bridge decks, sidewalks, and other surfaces. Several tests were performed using two sensors prototypes under different surface conditions (e.g., dry, surface ice, frozen, and wet) to simulate the effect of weather conditions on sensor output. The resistance across two sets of stainless steel poles, embedded at the surface of the concrete sensor, was monitored to develop a decision algorithm based on measurements of surface temperature and electrical resistance. Laboratory results have shown that the sensor output could be an effective basis for detection and reporting of surface ice, frozen concrete, and wet conditions.
2. Two sets of poles were used: the LUS (Look-Up and Side) poles and LU (Look-Up) poles. Information obtained from both pole types were used to detect and confirm various conditions. Moisture presence drives the resistance (output voltage) lower, and the thickness of the ice layer affects the resistance (resistance decreases when the thickness of the ice increases). On the other hand, chloride contamination from deicing salt reduces the resistance of the concrete.
3. The results of several tests conducted on LUS and LU poles under frozen and surface ice conditions indicated that these two conditions are distinguishable. A decision algorithm based on the measured surface temperature and output voltage of both sets of poles has been developed. The findings of friction tests indicated that the most dangerous conditions (from the friction standpoint) are surface ice and a combination of frozen concrete and surface ice conditions.
4. The decision algorithm is designed to distinguish and identify surface conditions and to issue different warning levels accordingly. The proposed decision algorithm considers the effects of salt contamination on the roadway surface.
5. The developed sensor system would have two embedded Wheatstone bridge circuits, a long-term battery, a logic controller, and a local-area signal transmission capability. The signal could either be used for local site warning signal/lights/messages or be relayed to a transportation control center, displayed on a web site, or communicated to vehicle information systems. The sensor could also transmit coordinates of its location. The warnings could be received by drivers, transportations authorities, and vehicle control systems to warn drivers of surface ice formation.
6. Ultimately, the findings of this study could be used in many different fields and applications. The proposed sensor relies on low-cost and simple technologies that could be applied on a mass scale.

## Figures and Tables

**Table 1:** Voltage–temperature zones for various conditions.

Poles	Surface Condition	Surface Temperature (°C)	V_G_ (V)
LUS	SI	−15→−9	0→+1.25
		−9→0	−0.8–0.1T→+0.13–0.14T
	FR	−15→−8	−2.1–0.1T→0
		−8→0	−2.1–0.1T→0.8–0.1T
	W	0→40	−1.0→−2.0
	D	−15→40	+1.5→+2.0
LU	SI	−15→0	−1.0→+1.0
	FR	−15→0	+1.5→+2.0
	W	0→40	−1.0→−2.0
	D	−15→40	+1.5→+2.0
